# Applying Control-Value Theory and Unified Theory of Acceptance and Use of Technology to Explore Pre-service Teachers’ Academic Emotions and Learning Satisfaction

**DOI:** 10.3389/fpsyg.2021.738959

**Published:** 2021-11-08

**Authors:** Changcheng Wu, Xue Gong, Li Luo, Qingling Zhao, Shan Hu, Ya Mou, Bin Jing

**Affiliations:** ^1^School of Computer Science, Sichuan Normal University, Chengdu, China; ^2^Department of Laboratory and Equipment Management, Sichuan Normal University, Chengdu, China; ^3^Faculty of Artificial Intelligence in Education, Central China Normal University, Wuhan, China; ^4^Shuang Liu Middle School, Chengdu, China

**Keywords:** academic emotion, learning satisfaction, MOOC, CVT, UTAUT, learning interest, technology acceptance

## Abstract

Academic emotions refer to the emotions related to achievement activities or outcomes. Academic emotions are directly related to learning performance and have been recognized as critical to learners’ learning satisfaction and learning effectiveness in the online learning context. This study aimed to explore the relationship between academic emotions and learning satisfaction and their underlying mechanisms in massive open online courses (MOOCs) learning context using mediation models. This study adhered to the theoretical frameworks of the control-value theory (CVT) and the unified theory of acceptance and use of technology (UTAUT). Participants were 283 pre-service teachers who volunteered from a normal university in Southwestern China. Results revealed that: (a) academic emotions did not predict learning satisfaction; (b) learning interest and technology acceptance fully mediated the influence of academic emotions on learning satisfaction; (c) the four dimensions of technology acceptance did not mediate the relationship between academic emotions and learning satisfaction. This study integrated CVT and UTAUT models, and the results emphasized the importance of academic emotions and learning satisfaction in CVT and provision of additional support for UTAUT. Therefore, these findings have significant implications for improving the quality of MOOCs in the post-pandemic era.

## Introduction

In 2020, the coronavirus disease 2019 (COVID-19) resulted in a pandemic (Zis et al., 2021); thus, higher education was affected worldwide. Subsequently, all walks of life advocated home isolation and reduce outgoing to alleviate the spread of the virus. Most educational institutions worldwide have also been shut since March 2020 (Jiang et al., 2021). Accordingly, local governments have been encouraged to endorse online learning platforms through Internet education resources to ensure the health and safety of learners and prevent the spread of the pandemic to schools (Iosif et al., 2021). This phenomenon has forced many normal universities to switch to massive open online courses (MOOCs) (Ministry of Education of the People’s Republic of China, 2020). These technology-focused online learning environments play an important role in pre-service teachers’ learning. The academic emotions experienced in these contexts are pivotal for their cognitive and emotional development (Graesser, 2020). Many pre-service teachers have expressed that transitioning from normal university to student teaching or to teaching as a novice teacher can be an emotional and disturbing period (Hascher and Hagenauer, 2016). Thus, it is especially necessary to conduct further research on the academic emotions of pre-service teachers during MOOC learning amidst the COVID-19 pandemic.

The success of MOOC learning is typically evaluated through online learning satisfaction (Hew et al., 2020; Jiang et al., 2021). Some studies have suggested that learning satisfaction is correlated with strong intentions and willingness to participate in MOOCs (Al-Samarraie et al., 2018; Salam and Farooq, 2020), lower MOOC dropout rates (Hew et al., 2020), and improved learning performance (Al-Fraihat et al., 2020). Despite the heavy monetary investments for new technological aspects by MOOCs’ platform developers, learners are not fully satisfied due to barriers such as difficulty to achieve openness, lack of interactive communication, and poor learning experience (Jiang and Zhao, 2018; Jiang et al., 2021). Therefore, further investigation is required to identify the determinants of learning satisfaction. Previous studies mainly used cognitive learning performance as the evaluation index for MOOC instructing quality (Barajas and Gannaway, 2007). The field of higher education has conducted several studies on learning satisfaction at the emotional and psychological levels (Shen et al., 2013; Al-Samarraie et al., 2018; Jiang et al., 2021), with ordinary university students as participants. However, few scholars have performed research with the specific subsample of pre-service teachers. Chen and Sun (2020) examined the learning satisfaction of pre-service teachers in a Chinese normal university and reported that moderate learning satisfaction levels, with a lot of room for improvement. Moreover, learners demonstrated lowest satisfaction levels with the hardware facilities.

According to the control-value theory (CVT), academic emotions influence learners’ motivation to learn, their learning strategies, and self-regulated learning, thereby influencing their learning achievement (Pekrun, 2006). Existing studies in the field of education have also shown that learners’ learning is closely related to their academic emotions (e.g., Artino and Jones, 2012; Noteborn et al., 2012; Owens et al., 2014; Zu et al., 2021). However, existing literature on academic emotions is majorly focused on traditional face-to-face instruction modules, and studies on academic emotions during MOOC instruction modules amidst the pandemic are relatively limited.

Moreover, the effectiveness of implementing information technology or systems is determined through user acceptance (Davis, 1989; Chao, 2019). This criteria extends to learners’ perception of the MOOC platform during the pandemic. Venkatesh et al. (2003) developed the unified theory of acceptance and use of technology (UTAUT), which is a popular framework in technology acceptance research. UTAUT is an integrated model incorporating eight models and prominent theories, including the technology acceptance model (TAM), theory of reasoned action, and theory of planned behavior (Chao, 2019). It aims to predict or explain new technology adoption and facilitate the understanding of technology acceptance (Chao, 2019).

Therefore, this study aimed to address these study gaps and employed CVT and UTAUT models to further explore the relationship between academic emotions and learning satisfaction among pre-service teachers. The following section elaborates on the CVT and UTAUT models and their association.

## Theoretical Framework

### Control-Value Theory

**FIGURE 1:**
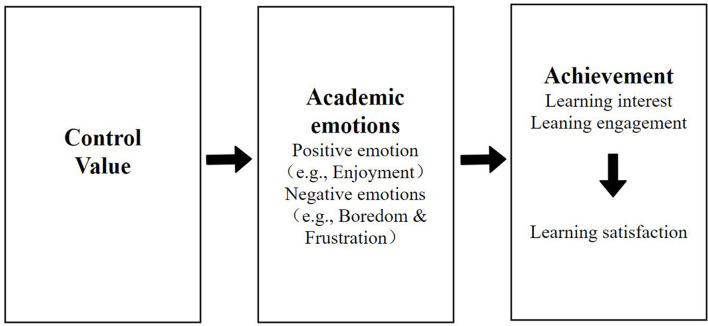
Control-value theory (Pekrun, 2006). A solid arrow shows the direction of the prediction.

MOOC learning is supplemented by intense emotional experiences (Yu et al., 2020). CVT proposes that academic emotions are emotions related to achievement activities or outcomes (Pekrun, 2006). CVT is a theoretical framework that examines the relationship between academic emotions and learning satisfaction (Pekrun, 2006). Academic emotions are learner’s feelings associated with their learning process and outcomes. The learning process involves learners’ relatively stable and long-term emotional states and their complex subjective experience (Pekrun, 2006). Accordingly, CVT acts as an integrative framework to analyze the underlying causes and consequences of emotions experienced within achievement and academic contexts (Pekrun, 2006). Although control (i.e., expectations that persistence at studying can be enacted, and that it will lead to success) and value (i.e., the perceived importance of success) are the direct antecedents of academic emotions, we primarily emphasize the latter part of the framework (i.e., emotion and learning + achievement), while focusing on the relevant theory (Figure 1). Academic emotions are classified as positive activating (e.g., enjoyment), negative activating (e.g., frustration), and negative deactivating (e.g., boredom) (Pekrun, 2017) according to valence (i.e., positive and negative) and activation (i.e., physiologically activating states and deactivating states). However, this study primarily focuses on the distinction between positive and negative emotions (i.e., valence) (Pekrun, 2017). Several existing studies have employed the CVT framework and explored the control and value antecedents of pre-service teachers’ emotions (e.g., Hascher and Hagenauer, 2016; Stephan et al., 2019; Jenßen et al., 2021). However, most of these studies consider pre-service teachers as “educators” in the teaching internship process. Particularly, there is a lack of research on pre-service teachers’ emotions in the field of “school education” (Stephan et al., 2019). Stephan et al. (2019) demonstrated that pre-service teachers who engage in face-to-face instruction module experience different emotions than those involved in MOOC learning. Pre-service teachers engaged in MOOCs reported higher boredom, anxiety, and anger, and lower enjoyment than those involved in face-to-face courses. Overall, they experienced more positive than negative emotions in teacher education courses. In line with these findings, it should be noted that MOOCs were introduced to teacher education before face-to-face courses. The novelty of online learning contexts for both learners and lecturers may have caused the inability to adapt to the new learning standards (Stephan et al., 2019).

In CVT, learning satisfaction is considered the “achievement” (Pekrun, 2006) and is defined as the learners’ perception regarding the curriculum, learning experience, and value of obtaining education from an educational institution (Ke and Kwak, 2013; Hew et al., 2020). Hennig-Thurau et al. (2001) found that quality of instruction and learning satisfaction are important factors in maintaining learning loyalty. Learning satisfaction is an integral outcome for learners, as it influences their motivation levels, which is an important psychological factor that impacts their learning (Bolliger and Martindale, 2004; Hew et al., 2020). Moreover, learning satisfaction is an important variable, as it shares a strong positive correlation with learners’ perceived quality of instruction in all learning contexts (Hew et al., 2020). In the field of education, learning satisfaction has become a critical topic of interest for evaluating learning performance, and it acts as an essential factor actualizing the learning goals (Al-Fraihat et al., 2020). Zu et al. (2021) showed that pre-service teachers’ positive activating emotions (e.g., enjoyment and pride) can significantly positively predict their blended learning satisfaction, while negative deactivating emotions (e.g., boredom) demonstrated no significant effects on blended learning satisfaction. The existing findings on negative emotions have been inconclusive. A previous study argued that negative emotions impact cognition and behaviors negatively (Owens et al., 2014), thus reducing learning satisfaction (Lee et al., 2021). Conversely, some studies suggested that negative emotions promote the usage of metacognitive strategies and positively predict learning performance (e.g., Artino and Jones, 2012; Noteborn et al., 2012). This phenomenon indicated that negative emotions may not reduce learning satisfaction.

Learning interest can be regarded as “motivation to learn” (Pekrun, 2006). It refers to the immediate emotional response to certain conditions and/or stimuli in the learning context, manifested in enthusiasm and participation; it also encompasses an intrinsic motivation to continue learning (Rotgans and Schmidt, 2011). Existing literature suggested that pre-service teachers are not very interested in participating in the *Educational Theory* course (Geng, 2009). However, the current literature lacks research on the factors influencing the learning interest of pre-service teachers. Additionally, studies on learning interest have indicated that changes in academic emotions may be crucial in generating and sustaining interest (e.g., Pekrun et al., 2002; Pekrun, 2005, 2017; Silvia, 2006; Nummenmaa and Nummenmaa, 2008). For example, when learners experience positive emotions in the learning process (e.g., enjoyment and contentment), their interest is peaked. Conversely, negative emotional experience (e.g., boredom and frustration) reduce their interest (Nummenmaa and Nummenmaa, 2008; Pekrun, 2017). Many researchers have also revealed that changes in learning interest will generate different learning outcomes, whereby higher learning interests produced improved learning outcomes (e.g., Artino and Jones, 2012; Guo et al., 2020). Additionally, evidence indicated that higher learning interest improved learning satisfaction and increased the intention to continue participating in MOOCs, thereby, reducing the MOOC dropout rate (e.g., Hong et al., 2016; Al-Samarraie et al., 2018; Tsai et al., 2018; Salam and Farooq, 2020). Similarly, Chang and Chang (2012) observed a strong association between learner’s motivation and learning satisfaction. Furthermore, Dziuban et al. (2013) highlighted that learner’s online learning interest can significantly predict their satisfaction with the learning system.

CVT can efficiently explain the relationship between learners’ academic emotions and engagement (Pekrun, 2016). In this study, learning engagement refers to the learners’ efforts toward achieving their desired goals during the learning process (Jung and Lee, 2018). Learning engagement emphasizes the importance of behavioral engagement (e.g., taking notes while watching instructional videos or peer discussion) in learning activities. Additionally, it shares positive associations with emotional engagement, such as learning interest or satisfaction (Fredricks et al., 2004; Wang et al., 2021). Moreover, pre-service teachers’ engagement within teacher education contexts is not only important for their own learning, but it may also influence their future teaching practice (Saini and Abraham, 2019). Carini and Kuh (2003) suggested that pre-service teachers with active and collaborative learning experiences are more likely to use similar methods in their own teaching practice. Furthermore, Cakir (2013) identified pre-service teachers’ learning motivation and perceived academic challenges as the most important predictors of learner engagement. However, few existing studies have explored the causes and consequences of pre-service teachers’ learning engagement during learning activities. Currently, educators face one of the major challenges of creating a positive learning environment within the classroom, with the aim of increasing learner participation and reducing dropout rates (Gao et al., 2020), since learning satisfaction is closely associated with dropout rates (Hew et al., 2020). Therefore, we believe that learning engagement is closely related to learning satisfaction. In addition, academic emotions are considered to be key predictors of learner engagement (e.g., Fredricks et al., 2004; Pekrun, 2016; Garn et al., 2017). Evidence has demonstrated that positive emotions promote learning engagement, while negative emotions do not (e.g., Pekrun et al., 2010, 2011; Owens et al., 2014; Zhen et al., 2017). Accordingly, learners with more positive emotions are more likely to participate in learning, while learners who experience negative emotions are more likely to disengage in the learning process (King and Gaerlan, 2014). However, Turner and Schallert (2001) found that for some learners, some negative emotions, such as shame, can also increase learners’ learning motivation and prompt learners to change their learning behaviors. The exact impact of academic emotions on learning engagement is yet to be explored. Similarly, the relationship between learning engagement and satisfaction is also controversial. A previous study revealed that learning satisfaction can be improved through active learning, group discussion, and other learning engagement behaviors that can induce higher learning experience and reflection (Fisher et al., 2018). However, Luo et al. (2019) held opposing views and believed that learning engagement levels cannot be used to predict learning satisfaction. Thus, the predictive paths from academic emotions to learning engagement and from learning engagement to learning satisfaction are still debated widely and require further research.

### Unified Theory of Acceptance and Use of Technology

Teo et al. (2008a) used the technology acceptance model (TAM) to explore how perceived ease of use and utility, as well as subjective norms and facilitating conditions as external variables, predict pre-service teachers’ attitudes toward computer technology usage. The study revealed perceived usefulness as the strongest predictor of attitudes toward technology use. Additionally, Teo et al. (2008b) demonstrated that perceived usefulness, perceived ease of use, and computer attitudes are important determinants of pre-service teachers’ behavioral intention to use, which can be identified by exploring technology acceptance. In this study, technology acceptance is defined as pre-service teachers’ acceptance of the rain classroom MOOC learning platform (an artificial intelligence instruction tool divided into computer and mobile terminals, mainly used in higher education fields, within universities to deliver MOOC instruction in China) (Wang, 2017). Previous empirical findings have displayed that as an extension of TAM, UTAUT is the most effective model for analyzing technology acceptance (Venkatesh et al., 2003; Chao, 2019). Recently, more and more information technologies have been widely adopted to complement higher education. These technology-oriented contexts play an important role in MOOC learning. For instance, the rain classroom platform has been widely used by Chinese universities to complement MOOCs and blended learning models. Moreover, amidst the COVID-19 pandemic, it has provided free and efficient online instruction opportunities to millions of university learners and teachers (Jiang et al., 2021).

The UTAUT model contains four latent variables—performance expectancy (i.e., perceived utility of the rain classroom), effort expectancy (i.e., perceived difficulty of using the rain classroom), social influence (i.e., the effect of instructor or peer’s opinion on individual behavior), and facilitating conditions (i.e., learners have the required resources and knowledge to use the rain classroom) (Venkatesh et al., 2003; Zhou, 2011). In addition, it contains two dependent variables—behavioral intention to use the system and usage behavior (Venkatesh et al., 2003). Furthermore, technology usage is found to be moderated by gender, age, experience, and willingness to use (Venkatesh et al., 2003). Despite the wide acceptance of the UTAUT model, doubts exist about its ability to elucidate individuals’ technology acceptance (Chao, 2019). Some researchers have suggested that the UTAUT model’s predictive ability for technology acceptance can be enhanced by increasing the number of external variables (e.g., Zhou, 2011; Lee et al., 2017; Al-Samarraie et al., 2018; Chao, 2019; Chea and Luo, 2019; Lu et al., 2019). Thus, the original UTAUT model has been extended. Some researchers have incorporated perceived enjoyment (i.e., perceived pleasure and enjoyment of using the rain classroom) (Lee et al., 2017; Chao, 2019). Recent research has incorporated perceived enjoyment into the UTAUT model and found that it can be used as an antecedent of performance and effort expectancy (Lee et al., 2017). Prior studies also demonstrated a relationship between the dimensions of technology acceptance and learning satisfaction. For example, perceived enjoyment and effort expectancy can significantly influence learning satisfaction (Zhou, 2011; Chao, 2019; Lu et al., 2019), whereas performance expectancy and social influence cannot significantly predict learning satisfaction (Zhou, 2011; Al-Samarraie et al., 2018).

### Relationship Between Control-Value Theory and Unified Theory of Acceptance and Use of Technology

CVT model was developed upon analyzing the causes and consequences that influence academic emotions within achievement and academic contexts (Pekrun, 2006), while the UTAUT model was based on the communications and information science approach (Zhou, 2011); however, there are some overlaps between these two perspectives. For example, both these theories focus on elucidating learning behaviors and learning activity outcomes. Zhou (2011) extended the UTAUT model by introducing learning satisfaction. The research findings revealed that the dimensions of technology acceptance predict learning satisfaction (Zhou, 2011). Similarly, CVT also proposes the certain antecedents of learning satisfaction (e.g., self-regulation, motivation, emotion, and environment) (Pekrun, 2006). Therefore, both these models may partially predict similar results but using different perspectives.

CVT and literature review specifically indicated that learning interest and engagement directly impacted learning satisfaction. Academic emotions can also directly or indirectly influence learning satisfaction. Meanwhile, academic emotions can directly predict learning interest and engagement. Moreover, UTAUT and some existing studies that the dimensions of technology acceptance may influence learning satisfaction. In addition, an important feature of the UTAUT model is that it can be extended by introducing external variables to enhance its predictive ability. Prior information technology studies have attempted to integrate emotion-related constructs (e.g., perceived enjoyment, computer playfulness, and emotional usability) (Lee et al., 2017; Chea and Luo, 2019). However, these studies have not adequately focused on emotions. Furthermore, these emotion-related constructs are measures of emotional responses to the relevant technologies and do not address individuals’ core emotional experiences. Thus, Chea and Luo (2019) incorporated personal emotional experiences to the UTAUT model for the first time to boost its robustness. However, their research was conducted in a laboratory with a small sample size (*n* = 67). Therefore, their study findings may not be suitable for generalization.

Consequently, despite the distinct origins and unique terminologies adopted by CVT and UTAUT, these perspectives complement one another and may supplement explanations regarding the relationship between the academic emotions and learning satisfaction among pre-service teachers in the MOOC learning context.

### The Present Study

The current study integrates CVT and UTAUT to develop a mediating model. This research primarily aims to extend previous study findings by examining the relationship between pre-service teachers’ academic emotions and learning satisfaction in-depth amidst the COVID-19 pandemic.

Subsequently, we proposed the following hypotheses:

H1: Positive and negative emotions will positively and negatively predict learning satisfaction, respectively.

H2: Learning interest, learning engagement, and technology acceptance will mediate the effects of academic emotions on learning satisfaction.

H3: The four dimensions of technology acceptance will mediate the effect of academic emotions on learning satisfaction.

## Materials and Methods

### Context

The context of this study was the “*Computer Science Fundamentals*” online course. This course taught basic computer knowledge skills, which are necessary for normal university pre-service teachers. Additionally, this course played an important role for pre-service teachers to master appropriate information education methods in the era of information technology and formed certain educational abilities. The entire course was broadcasted live by the instructor. Each course module contained instructional videos, learning forums, assignments, and tests. The rain classroom was used as an instruction platform for the MOOCs. Pre-service teachers were allowed to post messages on the screen and in the discussion area and respond to messages in real-time. Furthermore, data regarding the learning status for pre-service teachers was specifically recorded in real-time and could be exported by the backstage of the learning platform.

### Participants

Participants were 283 pre-service teachers (195 females) from a normal university in Chengdu, Sichuan Province, in Southwestern China. Their ages ranged from 17 to 24 (*M_age_* = 18.96, *SD*_*age*_ = 0.86) years. Of these, over 70% were experienced in using the rain classroom. They belonged to five different majors.

### Data Collection

Data were collected online in May 2020. A researcher uploaded the questionnaire to WJX^1^ —an online survey tool. All participants attended the Computer Science Fundamentals course taught by one of our researchers, where they were invited to participate in this research. During the class, participants were informed regarding the study purpose and a researcher distributed questionnaires to them. Participants voluntarily and anonymously completed this online questionnaire in approximately 10 min.

### Instruments

#### Academic Emotions Measurement

Data were collected using the adapted version of Achievement Emotions Questionnaire (AEQ). Pekrun et al. (2011) develop the AEQ based on CVT (Pekrun, 2006). We selected three dimensions of AEQ to evaluate pre-service teachers’ academic emotions in this study—enjoyment (4 items, e.g., “I am enjoying the online course,” α = 0.88), boredom (5 items, e.g., “I feel bored while studying the online course,” α = 0.95), and frustration (4 items, e.g., “I feel very frustrated when studying the online course,” α = 0.95). Enjoyment was classified as a positive emotion, while boredom and frustration as negative emotions (Pekrun, 2017). All the items were rated using a 5-point Likert-type scale (1 = *strongly disagree* to 5 = *strongly agree*). The overall internal consistency (α) coefficient of this questionnaire was 0.84. For each dimension, the total score was the average of all the item scores across that dimension.

#### Learning Interest Measurement

In this study, we used the adapted version of the Learning Interest Questionnaire developed by Rotgans and Schmidt (2011). Moreover, pre-service teachers’ learning interest was evaluated using two dimensions of this questionnaire with two items each: attention focus (“I am fully focused in this online course” and “I am not distracted by other things,” α = 0.59) and subjective emotion [“I enjoy the topic of this online course” and “Presently, I feel bored (reverse scored),” α = 0.72]. All items were rated using a 5-point Likert-type scale (1 = *strongly disagree* to 5 = *strongly agree*). The overall internal consistency (α) coefficient of this questionnaire was 0.82. For each dimension, the total score was the average of all the item scores across that dimension.

#### Technology Acceptance Measurement

Data were collected using the adapted version of the Technology Acceptance Questionnaire developed by Venkatesh et al. (2003). Pre-service teachers’ attitudes and acceptance toward MOOC instruction were evaluated using four selected dimensions: perceived enjoyment (7 items, e.g., “Learning on this online course platform is a pleasant thing,” α = 0.97), social influence (3 items, e.g., “The instructor encouraged me to use this online course platform to learn,” α = 0.88), effort expectancy (4 items, e.g., “It’s easy for me to use this online course platform proficiently,” α = 0.88), and performance expectancy (4 items, e.g., “Using this online course platform to learn has improved my learning efficiency,” α = 0.96). All items were rated using a 7-point Likert-type scale (1 = *strongly disagree* to 7 = *strongly agree*). The overall internal consistency (α) coefficient of this questionnaire was 0.95. For each dimension, the total score was the average of all item scores across that dimension.

#### Learning Engagement Measurement

The backstage learning data of the rain classroom were recorded and exported to examine the learning engagement of pre-service teachers across three aspects. It included the total number of slides viewed, the frequency of check-in into the class, and the frequency of reading the bulletin board. The total score for learning engagement was the average score across after adding all three categories.

#### Learning Satisfaction Measurement

Data were collected using the adapted version of the Chinese-language Learning Satisfaction Questionnaire developed by Yang (2014). Pre-service teachers’ learning satisfaction was evaluated using three dimensions in this questionnaire: instructor instructing (2 items, e.g., “Overall, I am satisfied with the instructing of this online course,” α = 0.91), teaching content (5 items, e.g., “The learning content in this online course attracted me and helped me to learn,” α = 0.86), and learning context (5 items, e.g., “Overall, I am satisfied with the learning context and equipment for this online course,” α = 0.86). All items were rated on a 5-point Likert-type scale (1 = *strongly disagree* to 5 = *strongly agree*). The overall internal consistency (α) coefficient of this questionnaire was 0.94. The overall total score was the average across all item scores, while the total score for each dimension was the average of all item scores in that dimension.

### Data Analysis

All statistical analyses were performed using SPSS 22.0 and Mplus 8.3 software. First, we computed descriptive statistics and Pearson’s correlation coefficients for all variables and their corresponding relationships. Second, mediating effects were analyzed by standardizing all scores (Z-Score) and performing structural equation modeling (SEM). Third, DiCiccio and Efron (1996) recommended implementing a minimum of 2,000 replicates while performing Bootstrap analysis; however, in this study, we used 5,000 replicates to improve the estimation, but it required more computing time (Banjanovic and Osborne, 2016). In the current study, statistical significance was set at *p* < 0.05. Additionally, Pekrun (2018) suggested that learners’ academic emotions will demonstrate significant gender differences. Therefore, gender was controlled for as a covariate in the analyses; it was coded 1 = male, 2 = female.

## Results

### Descriptive Statistics and Correlation Analysis

Table 1 presents the mean, standard deviation, and correlation coefficients for all variables.

**TABLE 1:** Descriptive statistics and correlation analysis of each variable.

**Variable**	**1**	**2**	**3**	**4**	**5**	**6**	**7**	**8**	**9**	**10**	**11**	**12**	**13**	**14**	**15**	**16**	**17**	**18**
1. Enjoyment (Positive emotion)	1																	
2. Boredom	−0.34**	1																
3. Frustration	−0.29**	0.76**	1															
4. Attention focus	0.45**	−0.46**	−0.40**	1														
5. Subjective emotion	0.47**	−0.46**	−0.41**	0.75**	1													
6. Perceived enjoyment	0.53**	−0.36**	−0.25**	0.52**	0.53**	1												
7. Social influence	0.43**	−0.27**	−0.24**	0.51**	0.41**	0.64**	1											
8. Effort expectancy	0.45**	−0.26**	−0.22**	0.52**	0.49**	0.69**	0.78**	1										
9. Performance expectancy	0.55**	−0.38**	−0.28**	0.55**	0.55**	0.84**	0.66**	0.71**	1									
10. Instructor instructing	0.46**	−0.35**	−0.31**	0.53**	0.55**	0.59**	0.58**	0.62**	0.60	1								
11. Teaching content	0.51**	−0.40**	−0.33**	0.55**	0.59**	0.71**	0.64**	0.69**	0.71**	0.84**	1							
12. Learning context	0.52**	−0.38**	−0.30**	0.51**	0.52**	0.69**	0.59**	0.64**	0.69**	0.68**	0.78**	1						
13. Negative emotions	−0.34**	0.94**	0.94**	−0.46**	−0.47**	−0.33**	−0.27**	−0.26**	−0.35**	−0.35**	−0.39**	−0.36**	1					
14. Learning interest	0.49**	−0.50**	−0.43**	0.93**	0.94**	0.56**	0.49**	0.54**	0.59**	0.58**	0.61**	0.55**	−0.49**	1				
15. Learning engagement	0.11	−0.12*	−0.13*	0.14*	0.14*	0.07	0.16**	0.15*	0.13*	0.11	0.12*	0.15*	−0.13*	0.15**	1			
16. Technology acceptance	0.55**	−0.36**	−0.28**	0.59**	0.56**	0.90**	0.86**	0.88**	0.91**	0.67**	0.77**	0.74**	−0.34**	0.61**	0.14*	1		
17. Learning satisfaction	0.54**	−0.41**	−0.34**	0.58**	0.60**	0.72**	0.66**	0.71**	0.72**	0.91**	0.94**	0.90**	−0.40**	0.63**	0.14*	0.79**	1	
18. Gender	0.11	−0.18**	–0.09	0.15*	0.05	0.08	0.18**	0.08	0.10	–0.02	0.04	0.10	−0.14*	0.11	0.06	0.12*	0.05*	1
*M*	3.67	2.24	2.06	3.77	3.85	5.13	5.63	5.55	5.32	4.30	4.19	4.11	2.15	3.81	13.84	5.41	4.20	1.69
*SD*	0.80	0.97	0.99	0.76	0.78	1.23	1.03	0.99	1.19	0.62	0.54	0.67	0.92	0.72	3.52	0.99	0.56	0.46

M, mean; SD, standard deviation; *p < 0.05; **p < 0.01. Convert all data to a standardized form (Z-Score).

The results revealed significant positive correlations between positive emotion (i.e., enjoyment), learning interest, learning engagement, technology acceptance, and learning satisfaction (0.14 < *rs* < 0.79, *ps* < 0.05). Conversely, negative emotions (i.e., boredom and frustration) reported significant negative correlations with learning interest, learning engagement, technology acceptance, and learning satisfaction (−0.49 < *rs* < −0.13, *ps* < 0.05). Furthermore, significant positive correlations were also reported between positive emotion, perceived enjoyment, social influence, effort expectancy, performance expectancy, and learning satisfaction (0.43 < *rs* < 0.55, *ps* < 0.01). Negative emotions showed significant negative correlations with perceived enjoyment, social influence, effort expectancy, performance expectancy, and learning satisfaction (−0.40 < *rs* < −0.26, *ps* < 0.01).

### Assessment of Structural Equation Modeling Model

**FIGURE 2:**
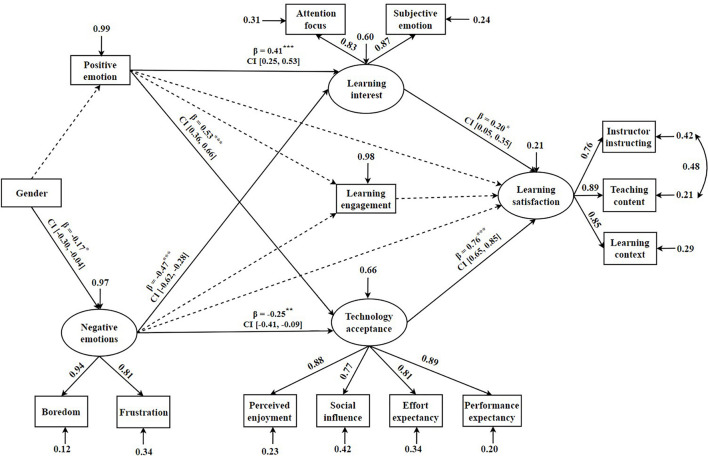
Mediation model for effects of academic emotions on learning satisfaction. The solid arrow represents the significant path, and the dotted arrow represents the insignificant path. β is the path coefficient. CI, confidence interval. *p < 0.05, **p < 0.01, ***p < 0.001.

This study implemented a mediation model (Figure 2) to examine the direct effects of academic emotions on learning satisfaction. To ensure conciseness in the model, all insignificant path coefficients and confidence intervals were deleted from the initial model. This model demonstrated a good data fit [*X*^2^/*df* = 3.71, Comparative fit index (*CFI*) = 0.93; Tucker-Lewis index (*TLI*) = 0.91, Root Mean Square Error of Approximation (*RMSEA*) = 0.09]. Figure 2 displays the hypotheses testing results for the direct and indirect path coefficients of this mediation model. The results suggested that there was insignificant direct effect of academic emotions on learning satisfaction. Moreover, positive and negative emotions were significant positive and negative predictors of learning interest (β = 0.41, *p* < 0.001; β = −0.47, *p* < 0.001, respectively) and technology acceptance (β = 0.53, *p* < 0.001; β = −0.25, *p* < 0.01, respectively), respectively. Furthermore, learning satisfaction was significantly positively predicted by learning interest (β = 0.20, *p* < 0.05) and technology acceptance (β = 0.76, *p* < 0.001). However, learning engagement was not predicted by academic emotions, and it did not predict learning satisfaction. Regarding gender, it significantly predicted negative emotions (β = −0.17, *p* < 0.05), revealing fewer negative emotions among females than males (Pekrun, 2018).

### Mediating Effects Analysis

The Bootstrapping method was employed to examine the direct and mediating effects of academic emotions on learning satisfaction (Table 2). We discovered that learning interest and technology acceptance fully mediated the effects of positive emotions (*g* = 0.08, *p* < 0.05; *g* = 0.40, *p* < 0.001, respectively) and negative emotions (*g* = −0.09, *p* < 0.05; *g* = −0.19, *p* < 0.01, respectively) on learning satisfaction. However, learning engagement did not report any mediating effect on the relationship between academic emotions and learning satisfaction.

**TABLE 2:** Bootstrapping analysis of the mediating effect test.

**Dependent variable**	**Independent variable**	**Mediating variable**	**Direct effect**	**Mediating effect**	**LLCI**	**ULCI**
Learning satisfaction	Positive emotion	Learning interest	0.04	0.08*	0.02	0.16
		Learning engagement	0.04	—	–0.004	0.02
		Technology acceptance	0.04	0.40***	0.28	0.53
	Negative emotions	Learning interest	–0.05	−0.09*	–0.20	–0.03
		Learning engagement	–0.05	—	–0.02	0.01
		Technology acceptance	–0.05	−0.19**	–0.31	–0.08

*LLCI, lower level of confidence interval; ULCI, upper level of confidence interval.*

*p < 0.05, **p < 0.01, ***p < 0.001.

### Assessment of Structural Equation Modeling Model (Four Dimensions of Technology Acceptance)

**FIGURE 3:**
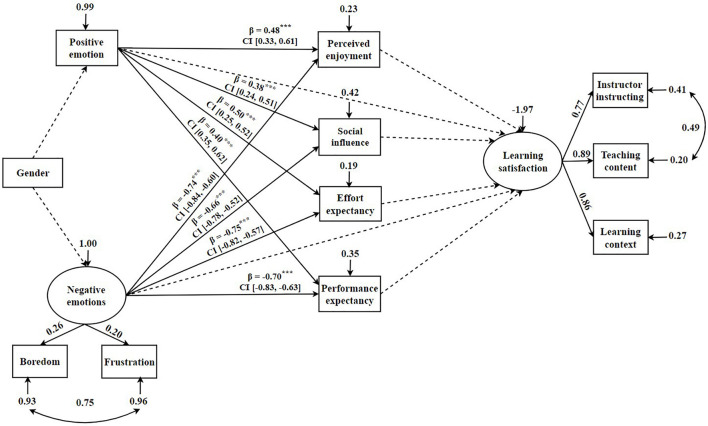
Mediation model for the effects of academic emotions on learning satisfaction through the four dimensions of technology acceptance. The solid arrow represents the significant path, and the dotted arrow represents the insignificant path. β is the path coefficient. CI, confidence interval. ***p < 0.001.

Technology acceptance was found to significantly mediate the relationship between academic emotions and learning satisfaction. Therefore, we developed another mediating model (Figure 3) to explore the effects of the four technology acceptance dimensions in depth.

Furthermore, we deleted the insignificant path coefficients and furthermore, we deleted the insignificant path coefficients and confidence intervals from this model. The model fit coefficients were: *X*^2^/*df* = 4.83, *CFI* = 0.94, *TLI* = 0.90, *RMSEA* = 0.12. Figure 3 displays the hypotheses testing results for the direct path coefficients of the mediation model. The results revealed that academic emotions did not predict learning satisfaction. Positive emotions significantly positively predict perceived enjoyment (β = 0.48, *p* < 0.001), social influence (β = 0.38, *p* < 0.001), effort expectancy (β = 0.50, *p* < 0.001), and performance expectancy (β = 0.40, *p* < 0.001). Moreover, negative emotions demonstrated a significant influence on perceived enjoyment (β = −0.74, *p* < 0.001), social influence (β = −0.66, *p* < 0.001), effort expectancy (β = −0.75, *p* < 0.001), and performance expectancy (β = −0.70, *p* < 0.001). However, none of the four dimensions of technology acceptance predicted learning satisfaction. Similarly, academic emotions were also not predicted by gender.

## Discussion

Stupnisky et al. (2019) suggested that pre-service teachers may be particularly susceptible to emotions due to their academic and professional expectations. In addition, teachers are at a higher risk of job burnout during their early teaching period, and they eventually quit their job due to the high levels of negative emotions (Vesely et al., 2014); thus, we believed that it was pertinent to examine their emotions during their learning phase as pre-service teachers. This study offered additional evidence for CVT and supported UTAUT extension by highlighting the importance of academic emotions and learning satisfaction in CVT. We found that academic emotions were not direct predictors of learning satisfaction. Learning interest and technology acceptance mediated the relationship between academic emotions and learning satisfaction.

### Mediating Effects of Learning Interest and Technology Acceptance

This study extended the initial model tested by Chea and Luo (2019) and introduced academic emotions in CVT into the extended model as the antecedent variable of UATUT. Furthermore, we found that academic emotions had no significant predictive effect on learning satisfaction (rejecting H1). However, this study finding was not consistent with some previous study results (e.g., Pekrun et al., 2010; Artino and Jones, 2012; Gong et al., 2016). Conversely, the findings regarding the relationship between pre-service teachers’ negative emotions and learning satisfaction were consistent with that of Zu et al. (2021), which indicated that negative emotions cannot directly predict learning satisfaction. This finding may have emerged due to the complexity of academic emotions. For instance, negative emotions (e.g., boredom and frustration) are negatively correlated with cognitive engagement, learning strategy use, and learning performance amidst traditional face-to-face learning contexts (Pekrun et al., 2011). However, in the MOOC context, some negative emotions, such as frustration, may motivate learners to learn better and employ more learning strategies (Artino and Jones, 2012; Noteborn et al., 2012), thus exerting different influences on learning performance and satisfaction. Therefore, a simple linear correlation could not be established in the relationship between academic emotions and learning satisfaction.

We also observed that both learning interest and technology acceptance mediated the effect of academic emotions on learning satisfaction. However, learning engagement did not report a significant mediating effect (partially supporting H2). Accordingly, the more positive the pre-service teachers’ academic emotions, the higher their learning interest and technology acceptance, thereby improving their learning satisfaction. Similarly, the more negative their academic emotions, the lesser their learning interest and technology acceptance, which resulted in decreased learning satisfaction. The above findings verified the perspective offered by Pekrun et al. (2002); Silvia (2006), and Pekrun (2017), that academic emotions are closely related to learning interest. Academic emotions can generate and maintain the learning interest in educational content (Krapp, 2005). The current research results further confirmed the previous empirical findings (Nummenmaa and Nummenmaa, 2008). That is, interest in online learning was associated with positive emotional experience. In addition, Zhang et al. (2006) highlighted that learners with high learning interest will tend to display positive learning performance and high learning satisfaction. Chang and Chang (2012) observed that there is a strong association between learners’ motivation and their learning satisfaction. Similarly, Dziuban et al. (2013) reported that learners’ learning interest can significantly predict their satisfaction with the online learning system. Therefore, the current study results were consistent with all the abovementioned research conclusions.

The integration model demonstrated a significant mediating effect of technology acceptance. Subsequently, we explored the mediating effects of the four dimensions of technology acceptance (i.e., perceived enjoyment, social influence, effort expectancy, and performance expectancy). We found no significant mediating effects of these four dimensions of technology acceptance on the relationship between academic emotions and learning satisfaction (rejecting H3). However, academic emotions significantly predicted all four dimensions of technology acceptance. This finding is consistent with previous study results (Chea and Luo, 2019). Accordingly, the more positive pre-service teachers’ academic emotions, the higher their technology acceptance. Conversely, the more negative their academic emotions, the lower their technology acceptance. Furthermore, Chea and Luo (2019) proposed that academic emotions can enhance the predictive ability of UTAUT; this proposition confirmed our study results. However, numerous existing studies have demonstrated the strong influence of both perceived enjoyment and performance expectancy on learning satisfaction (e.g., Zhou, 2011; Chao, 2019), where they identified the factors promoting learning satisfaction; these findings were inconsistent with the current study results. Moreover, some studies have reported that effort expectancy had a significant impact on learning satisfaction, while social influence demonstrated no significant impact (e.g., Zhou, 2011; Al-Samarraie et al., 2018; Chao, 2019) but our results revealed that neither effort expectancy nor social influence promotes learning satisfaction. Therefore, further research is required to examine the relationship between technology acceptance and learning satisfaction.

Contrary to previous research results indicating that learners with positive academic emotions are more willing to exert efforts into learning and have higher learning engagement levels (e.g., Fredricks et al., 2004; Pekrun et al., 2010; King and Gaerlan, 2014; Pekrun, 2016; Garn et al., 2017; Zhen et al., 2017), our study suggested that positive emotions failed to stimulate learning engagement. This may be caused by the generic action tendency of positive emotions; thus, they did not generate specific actions (Guo and Wang, 2007). This phenomenon may have resulted in insignificant prediction of learning engagement. Negative emotions also did not predict learning engagement, demonstrating inconsistent results with previous research findings, which showed that negative emotions exert a negative influence on cognition and behaviors (e.g., Pekrun et al., 2011; Owens et al., 2014) and hinder learning engagement further (Zhen et al., 2017). Some scholars pointed out that within MOOC learning contexts, learners experiencing frustration will motivate themselves to learn successfully (e.g., Artino and Jones, 2012; Noteborn et al., 2012) and enhance learning engagement in the learning process. Similarly, contrasting Fisher et al.’s (2018) perspective but verifying Luo et al.’s (2019) finding regarding the lack of impact of degree of behavioral engagement on learning satisfaction, this study revealed that learning engagement did not predict learning satisfaction significantly. Moreover, the current study is novel because most existing research on learning engagement was conducted in face-to-face classrooms, while our study explores the online classroom with pre-service teachers as participants amidst the COVID-19 pandemic. Data regarding learning engagement was recorded on the learning platform itself. Simultaneously, the insignificant results could also be explained by the lack of expertise of instructors and learners for operating the platform.

### Education Implications

The current results supported the CVT and UTAUT models and have important implications for educators and researchers, who are interested in improving the learning satisfaction of MOOC learners. Teachers can particularly improve learners’ learning satisfaction by promoting positive emotions while reducing their negative emotions. Positive emotions can stimulate learners’ learning interest and their technology acceptance, which is conducive to improving their learning satisfaction. Additionally, this study confirms the important role of academic emotions in adopting technology. This paper also provides good practical insight for MOOC platform developers, recommending the integration of learners’ emotional aspects into the system design (Chea and Luo, 2019).

## Limitation and Future Work

This study has several limitations, which can be profitably addressed to stimulate future research. First, the evaluation dimensions of academic emotions are not adequately thorough. Future studies should consider including the arousal degree of academic emotions in the measures. Another consideration is to incorporate machine learning, eye-tracking, and electroencephalogram technology to detect the changes in learners’ academic emotions during their learning process (Guo et al., 2019). Second, learner engagement was measured using frequency records in this study. Further research should assess learning engagement levels through the quality of their engagement. Third, this study employed a limited sample size. In the future, longitudinal research with a larger sample size can facilitate more diverse data collection and further verify the predictive ability of various dimensions of technology acceptance in CVT.

## Conclusion

The current study revealed that academic emotions did not predict learning satisfaction directly, but indirectly predicted learning satisfaction through learning interest and technology acceptance. Accordingly, the higher the positive emotions of pre-service teachers, the higher their learning interest and technology acceptance, thereby improving their learning satisfaction; conversely, the higher their negative emotions, the lower their learning interest and technology acceptance, thereby reducing their learning satisfaction. However, upon further exploration we discovered that none of the four dimensions of technology acceptance reported significant mediating effects.

## Data Availability Statement

The original contributions presented in the study are included in the article/supplementary material, further inquiries can be directed to the corresponding author/s.

## Ethics Statement

Ethical review and approval was not required for the study on human participants in accordance with the local legislation and institutional requirements. The patients/participants provided their written informed consent to participate in this study.

## Author Contributions

CW contributed to the study’s conception and design. LL, QZ, and SH performed the material preparation and data collection. XG, YM, and BJ performed the data analysis. BJ, XG, and CW wrote the first draft of the manuscript. All authors commented on previous versions of the manuscript, read, and approved the final manuscript.

## Conflict of Interest

The authors declare that the research was conducted in the absence of any commercial or financial relationships that could be construed as a potential conflict of interest.

## Publisher’s Note

All claims expressed in this article are solely those of the authors and do not necessarily represent those of their affiliated organizations, or those of the publisher, the editors and the reviewers. Any product that may be evaluated in this article, or claim that may be made by its manufacturer, is not guaranteed or endorsed by the publisher.

## Footnotes

1
www.wjx.cn
